# ReaDDy - A Software for Particle-Based Reaction-Diffusion Dynamics in Crowded Cellular Environments

**DOI:** 10.1371/journal.pone.0074261

**Published:** 2013-09-11

**Authors:** Johannes Schöneberg, Frank Noé

**Affiliations:** Department of Mathematics, Free University of Berlin, Berlin, Germany; Universite de Sherbrooke, Canada

## Abstract

We introduce the software package ReaDDy for simulation of detailed spatiotemporal mechanisms of dynamical processes in the cell, based on reaction-diffusion dynamics with particle resolution. In contrast to other particle-based reaction kinetics programs, ReaDDy supports particle interaction potentials. This permits effects such as space exclusion, molecular crowding and aggregation to be modeled. The biomolecules simulated can be represented as a sphere, or as a more complex geometry such as a domain structure or polymer chain. ReaDDy bridges the gap between small-scale but highly detailed molecular dynamics or Brownian dynamics simulations and large-scale but little-detailed reaction kinetics simulations. ReaDDy has a modular design that enables the exchange of the computing core by efficient platform-specific implementations or dynamical models that are different from Brownian dynamics.

## Introduction

Which molecules interact at which place and in which sequence, in order to orchestrate a specific cellular function? Understanding the detailed spatiotemporal mechanisms behind cellular processes is one of the main topics in current biology. This topic is driven by recent experimental advances, e.g. in super-resolution microscopy, which permit proteins to be counted and individually located in a cell, and demonstrated the existence of complex multiprotein architectures [1] [2] [3] [4]. The detailed mechanism of signal transduction events, such as phototransduction [5] or neurotransmission [6], involves the spatial coordination of molecules on length scales of 1 to 1000 nanometers, within timescales ranging from microseconds to seconds. Despite the recent advances in experimentation, it is still impossible to directly see such processes in detail. Computational approaches are thus essential to model the localization, the dynamical motion and the reaction kinetics of macromolecules, and thus help to fill in the space- and timescales that are not directly resolvable experimentally.

We propose that a computational model should include the following features to realistically simulate signaling mechanisms in cells:

1. *Single particle resolution*: In typical conditions, a given protein type occurs with order of 1000 copies in the volume of a cell [7]. In some organelles, proteins occur with copy numbers between 1 and 100, sometimes with a surprisingly precise stoichiometry [8]. These facts suggest, that concentration-based approaches such as ODE and PDE approaches are often inadequate [9], [10], and that treating proteins and other signaling molecules as explicit particles, with a specific location in space, is both feasible and necessary.
2. *Diffusion*: Biomolecules can only interact when they can physically reach one another. In many fast processes, e.g. in signal transduction, the time required for the molecules to form encounters becomes limiting, either because diffusion is slow compared to the reaction rates, or because the accessible space is limited [11]. For such situations, classical systems biology approaches such as ODE’s and Gillespie are inadequate, as they assume all species to be well-mixed. The particle dynamics should be explicitly modeled, e.g. through diffusion.
3. *Interaction potentials*: Biomolecules are densely packed in the cell, resulting in macromolecular crowding, as impressively demonstrated by the groups of Elcock [12], Ellison [13] and Skolnick [14]. Such space exclusions may play a role at molecular scaffolds [15] and may even be used in an ordered way to control reaction pathways, e.g. in the rod cell phototransduction module [16]. Furthermore, there is much evidence that both specific and non-specific attractive interactions exist between macromolecules. These may lead to formation of clusters [17], metastable pre-complexes [18] and other types of co-localization [19], [20]. Such clustering and co-localization leads to an effective reduction of the search space for binding partners, and may be essential for efficient signaling. These facts suggest, that it is important to include interaction potentials that permit to model repulsion and attraction of particles.
4. *Cellular geometry*: Cells constitute reaction containers that preserve certain conditions and properties inside and separate them from the outside environment via a boundary. The same is true e.g. for cellular organelles or cellular vesicles. This leads to multiple compartments, within which molecules may be restricted to stay, or from which they are excluded by the boundary. Such compartmentalization may also have other structural reasons, e.g. membrane patches fenced in by the cytoskeleton [21]. Furthermore, the special shape and geometry of the boundaries may play a role, not only by leading to a compartmentalization of molecules but also by the formation of local density gradients [22]. For these reasons, a detailed and encompassing modeling of the cellular geometry is important.
5. *Reactions*: We use the term reaction to indicate a change of state of a molecule, including changes in the chemistry, conformation, or aggregation state. Proteins transmit information by changing conformations. Complex formation and dissociation between macromolecules are ubiquitous ingredients of cellular signal transduction. Enzymatic reactions are required for most biochemical pathways. These facts suggest, that a computational model of cellular signaling should implement reactions that allow the simulated particles to change their state, and particles to be both created and annihilated.

Recent reviews [23], [24] provide a detailed overview of reaction-diffusion models and software. Here, we only give a rough overview in order to position our software with respect to existing projects. These can be roughly characterized by belonging to one of two classes:

### 

#### Reaction kinetics approaches

There are many approaches towards solving generated subvolume-based approximations to the spatiotemporal chemical master equation of reactive particles, including GMP [25], GridCell [26], Lattice Microbes [27]–[29], MesoRD [30], SmartCell [31], Spatiocyte [32] and Virtual Cell [33]. Reaction-diffusion approaches that explicitly propagate particle positions in continuous physical space include Cell++ [10], ChemCell [34], E-Cell [35], FLAME [36], GFRD [37], Klann et al. [38], MCell [39], Rigdway et al. [13] and Smoldyn [40] (see [41] for a review article). These approaches offer features (1), (2), (4) and (5), and can simulate long, biologically relevant timescales, but are usually lacking interaction potentials and thus the ability to represent complex molecular structures and scaffolds.

#### Molecular- and Brownian dynamics approaches

Simulation codes like DESMOND [42], DL_POLY [43], Gromacs [44], Hoomd-Blue [45], LAMMPS [46], NAMD [47] and OpenMM [48] were developed to simulate the molecular equations of motion in detail (usually thermostatted Hamiltonian dynamics or Langevin dynamics), but can also be used to simulate Brownian dynamics of coarse-grained molecules. Brownian and Langevin dynamics packages such as BD_BOX [49], Browndye [50], Brownmove [51], UHBD [52] and Elcock et al. [53] are designed to simulate molecular diffusion and molecular interactions. Simulations of cytosol dynamics provide illustrative application examples [12], [14]. These approaches offer features (1), (2), (3) and (4), but are usually limited to short simulation timescales (below milliseconds) and lack reactions as the simulated particle types and their number stay constant throughout the simulation.

**Figure 1:**
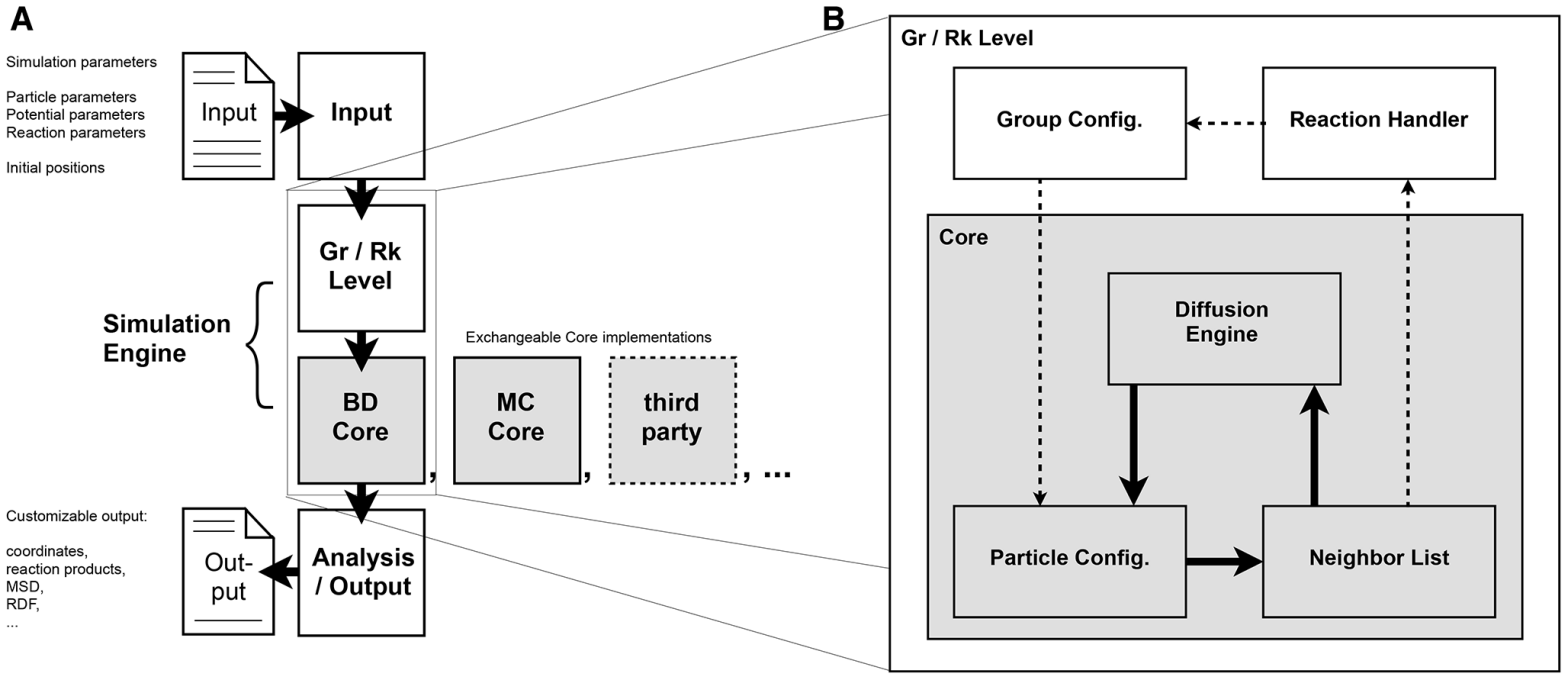
Workflow in Simulation and ReaDDy Code Design. **A:** Typical workflow and interplay between file input, file output and modules of ReaDDy. The left side of part A describes input and output functionalities of ReaDDy (sketched files) and how they interplay with code modules (squares). Among these modules, white drawn squares have access to both the particle level but also to information how particles are formed to groups. Grey squares are only based on particles to guarantee high computational efficiency. Modules communicate via interfaces, making them exchangeable. Currently two ReaDDy Core implementations exist, a Brownian dynamics based *BD Core* and a Monte Carlo based *MC Core*. The design is intended to encourage the incorporation of third party software to play the *Core*-role in the ReaDDy framework. **B:** Detailed view of the interplay between *Group/Reaction Module* (*Gr/Rk Module*), the *Core* module and their submodules during the main iteration loop. Most of the simulation time is spend on incrementing particle positions. As a result, the algorithm will circle between Particle Configuration, Neighbor List and Diffusion Engine (thick black arrows) to propagate diffusing particles. If a possible reaction event between two particle arises, this information is passed to the *Gr/Rk Module* module and is handled there before according changes of the Particle Configuration end that cycle (dashed arrows).

**Figure 2:**
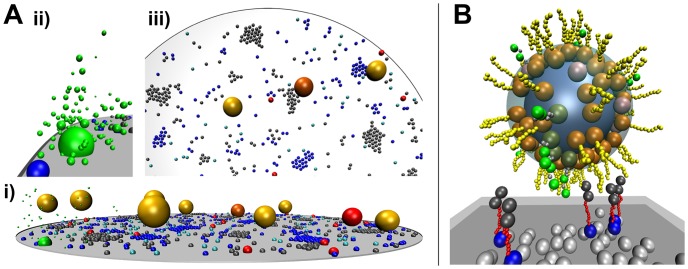
Possible Applications of ReaDDy at Different Levels of Modeling Detail. A model of vesicle fusion in the synaptic vesicle cycle is shown at two levels of detail. **A:** Snapshot of the simulation described in the ReaDDy tutorial. **i:** SNARE proteins syntaxin (blue), SNAP-25 (grey) and a calcium channel (green, large sphere) are modeled on a disk membrane, synaptic vesicles (yellow) float in the cytosol. Reactions allow the modeling of syntaxin’s conformational change (switch between light- and dark blue), the formation of SNARE complexes (red), vesicle tethering (yellow, orange and red vesicles, depending on the number of SNARE complexes involved) and calcium ion release (small green particles in panel **ii.** **iii:** short range attraction potentials induce clustering of SNARE proteins. **B:** Grouping of particles allows proteins to be modeled with complex shapes: syntaxins here consist of a membrane anchor (blue), a flexible peptide domain (red) and the Habc domain (dark grey). Synaptobrevin (orange and yellow) and synaptotagmin (dark green, grey, green) are also modeled as groups of particles, representing protein domains. Interaction potentials of plasma- (dark grey) and vesicle membrane (light blue) with anchor particles ensure, that membrane proteins can not leave the membrane.

In this paper we introduce ReaDDy (**Rea**ction **D**iffusion **Dy**namics). ReaDDy is a particle-based reaction-diffusion simulation package that is suited for crowded cellular environments and implements all features (1–5). It thus bridges the gap between current reaction kinetics and molecular dynamics approaches by combining a reaction engine with an explicit space and time simulator for particles, diffusing in a potential landscape. ReaDDy is based on an open architecture design that allows existing particle simulation packages to be included as modules (Fig. 1), and is thus meant as an expandable framework for simulations of cellular signaling. Its level of capturing molecular detail ranges from modeling molecules as single sphere particles to defining them as groups of spheres, held together by potentials (Fig. 2).

## Theory

The molecules or molecular domains represented in our reaction-diffusion model will henceforth be called “particles”. Particles are objects that have a size, a type and a position. Values of these attributes may change over time, especially due to reactions between particles. Particles may represent proteins, protein domains, ligands, lipids, ions or other biomolecules. When appropriate for the modeled signaling process, a particle may even correspond to a larger biological object, such as an entire vesicle or an aggregate of proteins. The theoretical foundation, governing the reaction-diffusion dynamics of and between these particles as it is used in ReaDDy, is laid out subsequently.

### Particle Diffusion

To obtain equations of motion of selected particles that represent the biomolecules of interest for the signaling process studied, one may invoke the Mori-Zwanzig formalism [54], [55], obtaining a generalized Langevin equation. Under the assumptions that (1) we reside in the over-damped limit [56], (2) memory effects from non resolved particles have died out on our timescales, (3) long-range density correlations (hydrodynamics [57]) average out between resolved and non resolved parts of the system and that (4) particles are treated as spheres, we arrive at the over-damped memoryless Langevin equation with isotropic diffusion, also known as isotropic Brownian dynamics (BD):
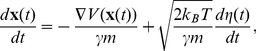
(1)where 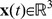 is here a 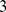-dimensional vector indicating the instantaneous position of a particle at time 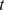. The change of the position over time 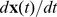 depends on the gradient (spatial derivative) of the potential 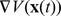, divided by the friction 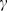 times the particle mass 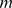. The resulting first term on the right hand side of Eq. (1) is the deterministic force. The second term on the right hand side is the stochastic force depending on the thermal energy 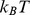, with Boltzmann constant 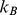 and temperature 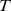. 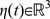 is a three-dimensional Wiener process, i.e. each component is an independent random process with normally distributed increments 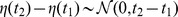. The fluctuation-dissipation theorem relates friction and temperature *via* the diffusion constant 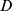:
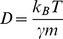
(2)and allows Eq. (1) to be rewritten as:



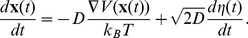
(3)The above equation may be solved numerically by employing an Euler discretization with constant time step 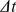, obtaining a discrete sequence of configurations in time, 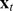, related by:
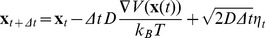
(4)where the noise is realized by independent normal variables 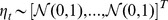. 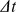 needs to be chosen shorter than the smallest timescale of the system, i.e. it depends on the stiffness of the potential and on the diffusion constants. When 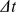 is sufficiently small, the Brownian Dynamics will, in absence of reactions, sample from the stationary distribution that is fully defined by the potential, Eq. (8). This fact is exploited in order to test the numerical correctness of the integration scheme (see section *Parametrization and Validation*).

### Reactions

Reactions are here understood as events which transform particles into other particles (which may also include the addition or deletion of particles upon a reaction). Reactions in the sense of the present model do not only encompass chemical reactions, but may also represent various physical processes, including conformational changes, aggregation events, etc.

We limit ourselves to uni- or bimolecular reactions, i.e. the types:
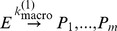
and



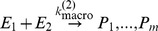
where 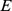, 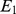 and 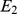 are “educts”, i.e. the particles that are consumed by the reaction and 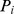 are the products, i.e. the particles that are created by the reaction (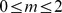). Reactions involving more than two educts can be modeled by splitting them up into multiple bimolecular reaction steps. The reaction rate constants, 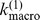 and 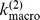, express the fraction of educts converted into products.

For unimolecular reactions this leads to the following set of ordinary differential equations [58]:
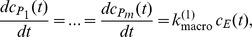
where 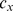 is the time-dependent concentration of particle type 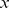. In unimolecular reactions, the reaction rate constant 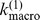 represents a single-molecule event - it measures the inverse mean time needed for the educt to decay into products. Thus these experimentally determined values are identical to the microscopic reaction rate constants used in our model description, 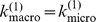.

The situation is more difficult for bimolecular reactions. The total rate, i.e. the total number of executions of the reaction per time unit of a bimolecular reaction taking place in a homogeneous reaction container is given by [58]:
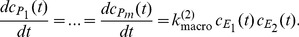
(5)with 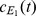 and 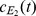 being the particle concentrations of educts and 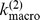 being the apparent rate constant. 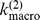 conceals many microscopic details. In particular, for a reaction event to occur, both 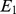 and 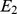 must first come close, forming an encounter complex. Subsequently, the chemical/physical reaction barrier will be overcome with a certain rate 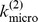, thus executing the reaction. The first step, the encounter formation, happens *via* particle diffusion, while the activation step is conceptually a first-order reaction of the encounter complex 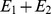 that reacts to the products:



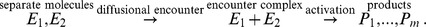



In a particle simulation, each particle’s location and diffusional motion is explicit. Therefore, encounter and activation processes have to be distinguished, as the motion leading to the encounter complex is directly simulated, and the activation can then only be conducted for those pairs of molecules that are close enough to form an encounter complex.

For each particle, a reaction radius 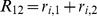 (see Table 1) is defined. When the inter-particle distance 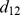 is smaller than 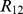, we have an encounter complex. The reaction radii are chosen based on physico-chemical intuition in order to represent a distance at which reaction partners are close enough such that their subsequent interaction is specific for this pair of molecules and can no longer be treated by a diffusion model in which the molecules may move independently. In other words, 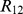 may be regarded as the distance at which the interaction between these two molecules becomes significant. Since electrostatic interactions are the most long-ranged, they are suitable to define 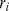. In cytosol, electrostatic interactions are negligible after 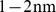, along membranes they can extend up to 4 nm. Changing 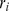 would change the encounter rate, and thus also change the activation rate needed to yield a given total reaction rate 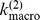. Thus it is clear that the separation of the total rate constant into encounter and activation rate constants is to some degree arbitrary, hence we use the convention that 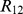 is fixed first, and the activation rate constant is determined subsequently.

**Table 1:** Particle Parameters and Resulting Properties of the Benchmark System.

	Type 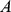	Type 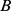	Type 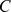
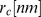	1.5	3	3.12
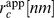	1.275	2.725	2.808
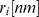	1.5	3	3.12
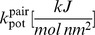	10	10	10
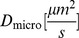	143.1	71.6	68.82

Parameters for particle types 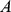, 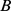 and 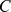. 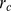: collision radius defining the onset of particle-particle repulsion. 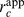: apparent collision radius that arises from both the collision radius 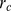 and the chosen inter-particle repulsion force constant 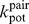. 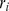: interaction radius for particle-particle reactions. 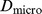: microscopic diffusion constant.

What is the activation rate constant 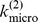 for a bimolecular reaction in 3D that is needed in our model to reproduce a measured total rate constant 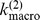 when a reaction distance 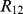 has been defined? Consider a homogeneous mixture of particles of types 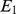 and 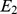, which freely diffuse with diffusion constants 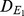 and 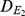, and form an encounter at distance 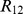. The rate at which encounter complexes at distance 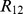 are formed is given by the Smoluchowski equation [59].
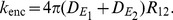
(6)


Now taking into account that educts, when having diffused into a distance of 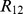, are not absorbed there entirely as in (6) but react to products with constant rate 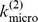, leads to the following equation (see [60] for derivation):
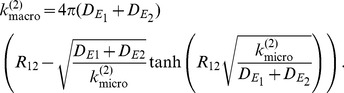
(7)


Please note that Eq. 7 only holds in 3D and that the derivation of the same relationship in 1D and 2D becomes more involved. See [61] for a thorough discussion of this subject, which goes beyond the scope of the present paper.

In the particle simulation, time is discretized into segments of 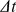. The reaction rate must therefore be converted into a probability that the reaction will take place in a given time step. When making the assumption that a single particle cannot undergo multiple reactions in one time step (which is only true if the time step is sufficiently small, compared to the reaction rates involved), the reaction probability is then obtained from the Poisson probability of finding at least one reaction event with rate 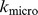 in a time window 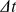 [62]:
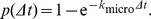



### Interaction Potentials and Stationary Distributions

Inter-particle potentials are useful for modeling space exclusion (e.g. crowding effects, cellular walls), for keeping particles in certain regions (e.g. diffusion on a membrane), for modeling particle aggregation, and for modeling the correlation of particle motions due to electrostatic interactions. The potential 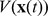 assigns a potential energy to a particle configuration 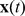, which has henceforth dimension of the joint space of all particle positions. Note, that both, the terms active in 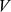 and the length of the position vector 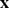, will change over time, because of particle reactions that change the particle composition of the system. However, in between two reaction events, 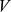 is unique and during this time, the potential has an associated stationary density given by
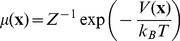
(8)where 
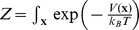
 is the partition function. In many real simulations, 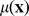 will not be sampled from because of the reactions taking place that drive the system out of equilibrium. However, 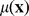 is useful to parametrize the particle interactions to the expected behavior in a stationary or quasi-stationary state. Since 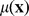 is a stationary property of 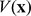, we can furthermore use it as a reference to evaluate the numerical correctness of the particle dynamics in the absence of reactions. 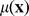 is useful to calculate all kinds of stationary properties, for example the radial distribution function (RDF) between particles of set 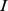 with those of set 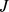, defined by the ensemble average:
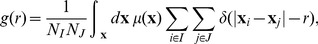
(9)where 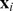 and 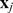 are the subvectors of 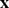 describing the locations of particles 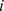 and 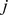. This density is in practice approximated with a histogram with bin size 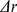, obtained from a set of configurations at time-steps 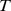:
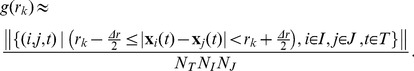
(10)where 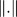 denotes the size of the set and 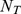, 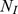, and 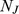 denote the number of time steps used and the sizes of sets 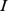 and 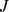, respectively. If the RDF is calculated for a system that is bounded by a box, a scaling factor [63] has to be applied in order to correct for boundary effects.

For our purposes we consider two types of potentials. With potentials of *order one*, we refer to potentials that depend on the coordinates of only one particle to determine the respective potential energy or displacement vector associated with it. These potentials are usually used as simulation geometry defining potentials, e.g. cellular walls. With potentials of *order two*, we refer to potentials that depend on the coordinates of two particles to determine the potential effects. These potentials are usually used for particle interaction potentials, e.g. softcore particle repulsion.

## ReaDDy Software

The implementation design of ReaDDy is sketched in the subsequent sections.

### Simulation Algorithm

Given an initial particle configuration 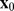, the simulation model propagates the positions and states of the particles involved in discrete time steps. In each time step, it performs two actions (1) a BD step of all particles in the potential 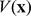 and (2) a reaction step that may change particle types and numbers. Finally, all changes accumulated during step (1) and (2) are executed, the pairwise distances between particles are updated and the current simulation time 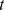 is incremented by 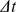 before a new cycle is started. The simulation algorithm can be summarized as given in Algorithm 1.

**Algorithm 1.** Pseudocode of the ReaDDy particle simulation algorithm:

1. Start with time 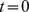, an initial particle configuration 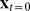

2. Repeat for 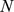 steps (total simulation time 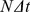):
  - Advance the Brownian dynamics by one step of length 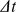, based on potential 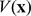 and the particle type dependent diffusion constants 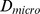

  - Create a list of reactions that can occur. For each particle that can react, choose a reaction with probability depending on its rate constant and execute the reaction with probability 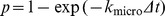.

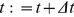
(c)The implementation of the algorithm is described subsequently.

### Software Architecture

The ReaDDy framework has been designed using the following principles:

1. *Modularity*: ReaDDy is intended to bridge the gap between BD and MD packages (including potentials, lacking reactions) on one hand and particle-based reaction-diffusion simulations (lacking potentials, including reactions) on the other. Some of these available packages are highly optimized and perform their tasks very efficiently. For this reason ReaDDy is designed to consist of exchangeable modules that could be replaced by efficient existing codes.
2. *Expandability*: ReaDDy follows an object oriented and interface based design. An interface abstraction exists for the entire simulator which renders all parts of ReaDDy to be easily interchangeable and expandable. ReaDDy is Open Source (BSD 3-Clause). Developers are invited to contribute additional module implementations. The available implementation is intended as a default that guarantees functionality without such additional modules.
3. *Platform independence*: The ReaDDy framework and the default module realizations are implemented in the Java programming language which guarantees that ReaDDy is fully functional on all computer architectures that support a Java runtime environment. This choice was made as Java code can be quickly developed and debugged compared to C or C++, while having similar performance [64], [65]. Note that additional module implementations may be in other programming languages such as C or C++, and would thus require platform-specific compilation. Such native module implementations may be included into the ReaDDy framework via the Java Native Interface (JNI).

ReaDDy consists of three main modules: The *simulation engine*, the *input* and the *output* module (see also Figure 1).

The ReaDDy ***simulation engine*** essentially consists of two submodules, the *Core Module* and a *Group/Reaction Module (short Gr/Rk Module).* In most cases, reaction events occur rarely compared to the advancement of every particle position in every time step. In order to facilitate platform-specific high-performance implementations of the most time-consuming computations, the *Core Module* has been split from the *Gr/Rk Module*.

The ***Core Module*** propagates point particles and recognizes reaction rules that may fire (i.e. when pairs of educts reside within the reaction radius). Reaction rule recognitions are reported to the Group/Reaction level to be handled there. The *Core* itself does not execute reactions, nor does it know of superstructures, such as particle groups. This design allows the *Core* to be replaced by other implementations, e.g. Langevin dynamics, dynamics including hydrodynamic coupling between particles, or high-performance CPU or GPU implementations based on existing codes such as OpenMM [66]. See section *Efficient Neighbor Calculation* in the Supporting Information (Text S1) for the current optimization status of the the *Core*. Currently, two *Core* implementations exist:

1. Brownian dynamics core (default): Implements the Euler-discretized Brownian dynamics.
2. Monte Carlo core: Implements the Markov chain Monte Carlo (MCMC) method for particle moves described in the Supporting Information.

The ***Group/Reaction Module*** handles logical groups of particles and executes reactions. Particle groups facilitate the efficient modeling of complex processes e.g. polymerization reactions. The resulting logical structures are eventually mapped to particles that are handled by the *Core*. See Figure 1B for the interplay between *Core* and *Gr/Rk Module*.

ReaDDy uses five different input types: global, particles, potentials, groups and reactions that are orchestrated by the ***input module***. It splits input information into an only particle related part for the *Core* and a general part for the *Gr/Rk Module*. All input files are defined in the XML format (Please see Supporting Information (Text S1, *Input File Organization*) for further information).

The ***output module*** of ReaDDy is based on a runtime analyzer scheme. The user can choose between different runtime analyzers for multiple purposes e.g. trajectory output, output of reaction events, output of the MSD or the output of the RDF which are available in different output formats (e.g. xml, xyz, csv). There is a special output format that allows trajectories to be displayed in VMD [67]. The ReaDDy standard output format is readable as input, allowing to run new simulations from trajectory frames.

### Performance

The MD packages discussed above [42]–[46], [66], [68] as well as many BD [69] and reaction-diffusion packages [28], [29], [70]–[72] provide parallel computation. The ReaDDy implementation presented in this work is a single-core CPU version, intended to be a reference implementation of our design. It already anticipates parallelization by separating the costly BD and potential-evaluation steps, the *Core Module*, from the *Group/Reaction Module*. Because of this and the interface abstraction layer, the *Core Module* default implementation may be replaced by an already parallelized third party BD particle integrator.

Please note that the choice of Java as implementation language does not impair the performance of the code. Although early Java versions performed poorly, current Java codes perform similarly to C or C++ in numerical applications [64], [65]. High-performance ReaDDy cores that rely on C or C++ based libraries such as CUDA or OpenCL can be implemented either via JNI or via the Java bindings for both libraries, JCuda and JOCL.

Because ReaDDy is to our knowledge the first program that combines molecular-dynamics type particle simulations with reactions, a benchmark test across different tools is currently not meaningful. It can be stated however, that the performance of ReaDDy is more comparable to MD and BD packages, that integrate the dynamics of interacting particles using short time steps, rather than to reaction-diffusion packages,that do not involve particle interaction potentials, like e.g. Smoldyn [40]. Such reaction-diffusion packages may be orders of magnitude faster for dilute systems, as the particle interaction potentials in ReaDDy are the computationally most demanding part. In our benchmark systems (see below), the evaluation of reactions only takes about 5% of the algorithm’s runtime. Therefore, if a user intends to simulate dilute systems that do not require interaction potentials, it is currently recommended to use reaction-diffusion packages specialized for that task.

### Runtime of the Algorithm

**Figure 3:**
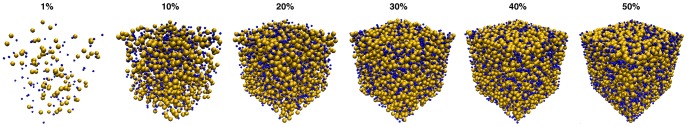
3D-Benchmark System Setups used in this Study. The occupied volume fraction ranges from 1% to 50% within a cube of 100 nm edge length. The 30% occupied volume fraction best resembles cytoplasm conditions.

**Figure 4:**
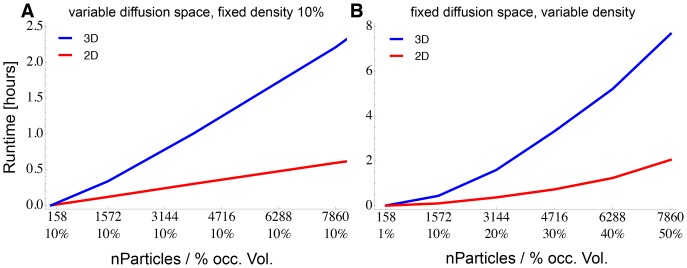
ReaDDy Runtime Benchmark. **A:** CPU time required (using single standard CPU cores) to run 100,000 simulation steps of benchmark particle systems at 10% occupied volume fraction with increasing system size and number of particles. Linear runtime can be observed. The blue curve represents a 3D container setup, the red line represents a 2D disk setup. **B:** Runtime performance for a fixed-volume simulation at different particle densities. Simulation volumes are a box of 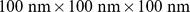 in 3D (blue, see Fig. 3 for illustration) and a disk of radius 297.4 nm in 2D (red). On average the density increase leads to a higher number of neighbors per particle and thus to a super-linear increase in runtime.

ReaDDy was benchmarked on the setups described in section *Parametrization and Validation* (see Figure 3) on an Intel Core i7 processors with 2.6 GHz. To illustrate the performance of the algorithm, we also included simulations on a two-dimensional membrane (particle coordinates had three dimensions but were held on the membrane by a potential). In a first test, the occupied volume fraction of the systems was fixed to 10%, and the simulation volume was adapted to the number of particles simulated. Fig. 4A reports the CPU time required to run 100,000 steps for these setups. It is seen that the runtime scales linearly with the number of particles. Integrating the dynamics of a 1000 particle system for one step takes 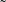
7.7 ms in 3D and 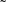
2.5 ms in 2D.

In a second benchmark (Fig. 4B), we kept the simulation volumes fixed to a 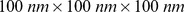 box in a 3D simulation, and a disk of radius 297.363 nm in a 2D simulation. Different particle numbers thus correspond to different particle densities. The runtime now scales quadraticaly with the number of particles, as a result of the increasing number of pairwise particle interactions per particle that have to be integrated when the system density increases. Note, that this increase of runtime is limited by the maximum occupied volume fraction that can be achieved with sphere packing, which is about 70% for equal sized spheres in 3D.

Table 2 gives an overview of the CPU times required to simulate 1000 particles for 1 ms (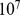 steps) in different simulation setups.

**Table 2:** CPU Runtimes to Simulate 1

	*p* = 10%	*p* = 30%
3D	21.43 h	44.34 h
2D	7.51 h	15.19 h
2D*	0.75 h	1.52 h

CPU runtimes in hours to simulate 1000 particles at densities of 10% and 30% occupied volume fraction in 3D box- or 2D disk geometry with 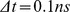 for 10,000,000 steps.

* 2D systems will likely represent membrane models of higher viscosity, usually resulting in one order of magnitude smaller diffusion constants. This enables the system to be integrated with a one order of magnitude larger timestep.

## Parametrization and Validation

### Benchmark System

To illustrate and validate the simulation methodology, we consider a benchmark system of two particle species 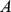 and 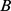 that diffuse and react in a quadratic container of edge length 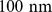 at a temperature of 20°C. To illustrate finite-size effects, the reaction container was equipped with repulsive walls rather than periodic boundaries. Our system includes the following second-order association and first-order dissociation reaction:
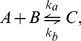
(11)with forward and backward reaction rates 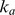 and 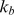. In order to model conditions similar to those found in cytoplasm, the collision and interaction radii (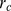,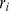) are chosen to be equal, and were set to 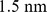 for particle type 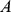 and 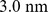 for particle type 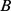, representing typical sizes of the most abundant macromolecules in cells. Radii 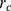 and 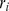 for particle type 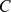 were set to 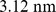, yielding the volume of 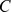 to be equal to the sum of the volumes of 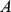 and 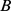 particles.

The microscopic diffusion constants 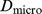 were calculated via the Stokes-Einstein-Equation
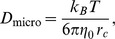
with 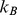 representing Boltzmann’s constant, *T* = 20°C the temperature and 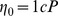 the viscosity of water. Here we used the water viscosity rather than the cytoplasmic viscosity because crowding effects are studied explicitly, i.e. all crowding particles are considered to be part of the benchmark system. As a result, we obtain the following microscopic diffusion constants for particle types 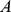, 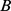 and 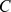: 
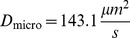
, 
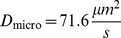
 and 
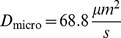
. See Table 1 for an overview of all particle parameters.

There are two potentials governing the dynamics of the system: First, a harmonic potential of *order one*, acting on every component 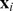 of the single particle positions 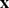, of the form
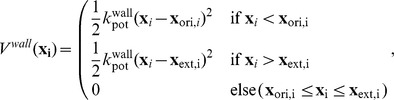
with the potential force constant 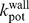 and the vectors 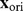 and 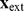 representing the lower left (origin) and the upper right corner (extension) of the container cuboid. The potential is designed such that particles within the container feel no potential but an escape over a boundary is penalized with a quadratic term. Second, a harmonic potential of similar form but of *order two*, acting on all pairs of particles 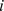 and 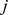, represents the particle-particle repulsion potential:



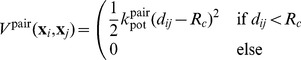
with the potential force constant 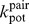, corresponding pairwise distances 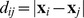 between particles 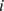 and 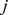 and the sum of the collision distances of the respective particles 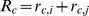. See the next section for information about the parametrization of these potentials.

Similar to [13], to investigate the influence of crowding, six setups of different particle density were used: 1%, 10%, 20%, 30%, 40% and 50%. For comparison: The experimentally determined value for cytosol of E. coli is known to be in the range 30%-34% [73]. See Table 3 for the number of particles and the molar concentration of the systems associated with the respective covered volume fractions and Figure 3 for an illustration of the density of the resulting systems.

**Table 3:** Particle Numbers and Particle Concentrations in Benchmark Systems.

Occupied volume fraction[%]	Total number of particles	Total molar concentration [ 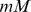 ]
1	158	0.095
10	1572	0.947
20	3144	1.893
30*	4716	2.840
40	6288	3.787
50	7860	4.733

Particle numbers and their concentrations for the different benchmark system setups in the box of 100 nm edge length.

* conditions similar to cytoplasm (compare Figure 3 for a visual illustration).

All simulations started from an equilibrated uniformly distributed initial configuration. Due to the space extension of the particles, pure uniformly distributed random configurations are likely to contain unfavorable overlaps between particles. Within the first few timesteps of a simulation based on such a configuration, these overlaps result in large repulsion forces between the involved particles. These forces cause particles to make large steps, which in turn is likely to produce other overlaps in the next timestep, causing the simulation to become unstable. To avoid this behavior, the particle configuration has to be relaxed first towards a low-energy state. We did this by applying the Monte Carlo (MC) algorithm described in Supporting Information (Text S1 and algorithm SA 1) for 3000 steps, prior to the BD simulation.

### Potential Parametrization and Validation of the Brownian Dynamics

The Brownian motion of particles depends on the particle interaction potentials and the diffusion constants. Besides the wall potential 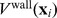, ensuring all particles to stay inside the simulation box, the co-localization of particles is here governed by the repulsive pair potential 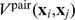 that prevents particles from penetrating each other and is thus the cause for crowding effects in the simulation.

**Figure 5:**
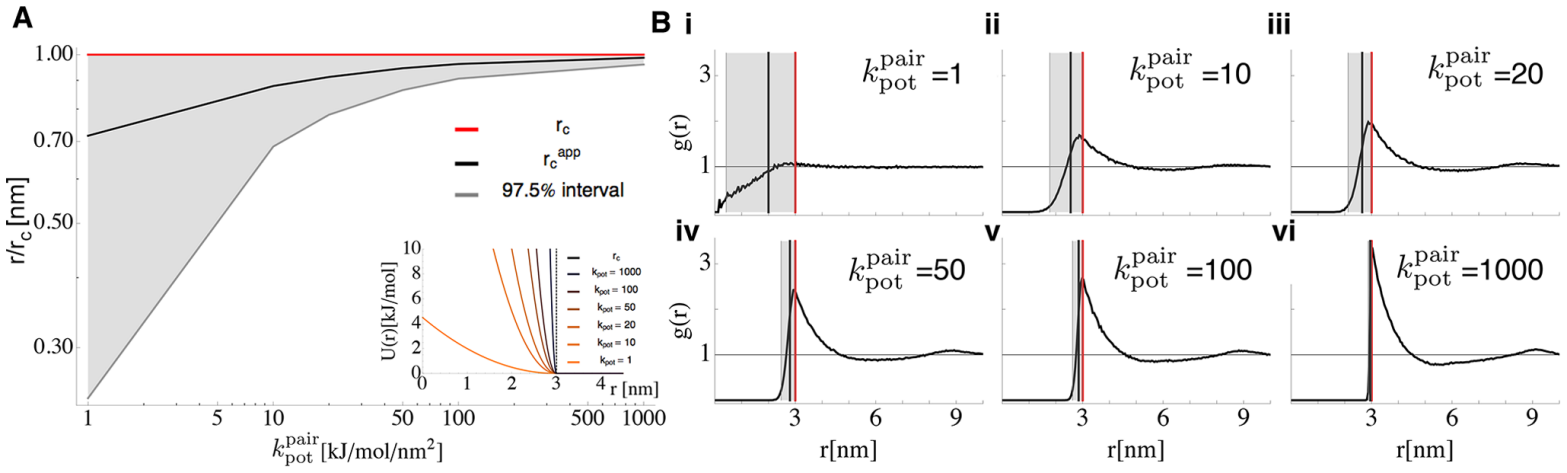
Apparent Particle Radii and Radial Distribution Functions (RDFs), Depending on Collision Radius and Potential Force Constant. Shown RDFs are based on particle-pairs of particle type 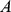 in the 50% occupied volume fraction benchmark system. **A:** smaller force constants 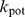 lead to larger overlap regions (grey area) and to larger differences between 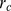 (red) and 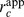 (black). The inset depicts the potential shape for different 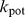. **B:** individual RDFs are depicted for different 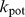 (i–vi). Same color code as in A.

In order to provide a guideline how inter-particle repulsion force constants should be chosen when using ReaDDy, we computed the radial distribution functions (RDF) for different force constants 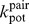 (Eqs. (9) and (10)). Fig. 5 reports these RDFs for a simulation setup with 50% occupied volume fraction, comprising of 3930 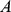 particles and 3930 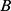 particles (see Table 3, setup 6). Since RDFs are purely stationary quantities, no explicit time-integration of the equations of motion is needed to calculate them. In order to avoid errors from time discretization, we have instead used the Monte Carlo algorithm described in Text S1. To measure the quality of the applied potentials in terms of resulting particle-particle overlaps, an apparent collision radius 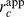 can be defined as follows:
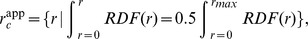
with 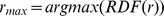. 50% of the area under the RDF from 0 to its first maximum lies left of 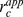 and 50% to its right. The difference between 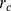 and 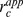 measures the overlap. Smaller potential force constants lead to larger overlaps. In a similar way, a 0.5% to 97.5% interval can be defined in which the 97.5% interval encloses the whole overlap region of the particles in the RDF. See Figure 5B for a depiction of this interval, 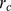, 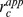 and their behavior for different force constants. The results suggest, that a force constant of 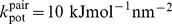 is a reasonable choice that both guarantees some spatial exclusion, while permitting some overlap in which reactions between particles 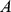 and 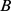 can occur. Hence, 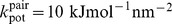 was used for all repulsion potential terms (including 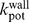).

### Brownian Dynamics Timestep Selection

**Figure 6:**
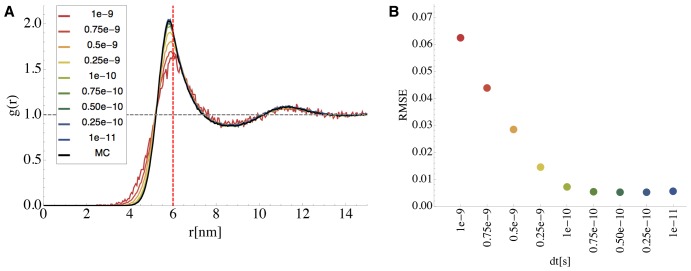
Determination of the Brownian Dynamics Time Step Length 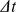. **A:** Dependency of the computed radial distribution function 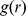 for different time step lengths 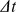. The black line shows the exact 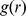 of 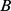-particles computed by Monte Carlo. The interaction potential was chosen to be a softcore repulsion potential (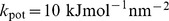) when their distance is closer than the sum of their collision radii 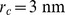. The colored lines show 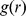
’s computed from time discretized Brownian dynamics simulations with different timesteps. **B:** Root mean square error of the difference between Monte Carlo derived g(r) and the discretized diffusion simulation (displayed in same color code as A).

For a given potential, the Brownian dynamics simulation time step 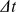 must be adjusted so as to limit the time-stepping discretization errors. This is because the time discretization (Eq. (4)) is a local linearization of the nonlinear potential, which is only a good approximation when the time step is small enough compared to the curvature of the potential. Thus, stiffer potentials require shorter time steps. Here, we adjusted the time step such that the Brownian dynamics simulation yielded radial distribution functions that matched those computed by the Monte Carlo algorithm (See Text S1). Fig. 6 shows a comparison of the 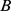
–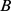 radial distribution functions 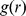 (see Eq. (9) and (10)) calculated with the Monte Carlo algorithm, and the Brownian dynamics discretization using different time step lengths between 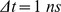 and 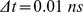. It is visible that smaller time steps result in a better approximation quality, finally reaching a plateau where discretization errors are small and the total error is dominated by statistical errors only (See part B of Fig. 6). We chose the largest timestep of that plateau, 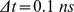, which was used henceforth. Significantly larger time steps resulted not only in differences of the radial distribution function, but also in undesirable dynamical behavior and a destabilization of the simulation.

### Diffusion and Crowding

**Figure 7:**
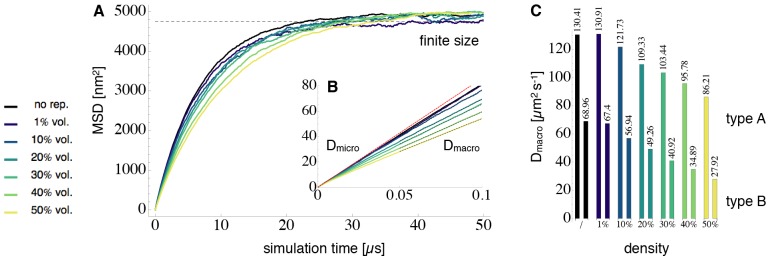
Mean Square Displacement (MSD) and Diffusion Constants for Particle Type 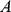 in the Benchmark System. In finite-sized systems, the MSD over time (thick colored lines, lighter color for denser system density) showed a triphasic behavior. **A:** On long timescales, the MSD can only reach a bound set by the finite system size (dashed black line). **B:** On short timescales it is visible that all curves share the same microscopic diffusion constant 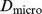 (dashed red line). In a setup where repulsion potentials between particles were switched off (thick black line), particles were only subjected to boundary repulsions and therefore remained diffusing closely to 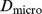. On intermediate timescales, particles in denser simulations including repulsion potentials, diffused according to a smaller apparent diffusion constant 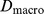 (dashed black fits). The higher the occupied volume fraction and the stronger the crowding, the smaller 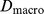. **C:** 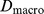 values for particle types 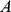 and 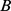, obtained from linear fit of the second linear phase of the curves in B.

Using the potential parameters and time step described above, the benchmark system was first simulated without reactions. Fig. 7A and 7B show the mean square displacement (MSD) of 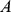 particles. On short timescales 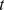, the particles in the simulation setup had a MSD of 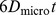, i.e. they exhibit normal diffusion governed by the equation 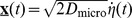 with diffusion constant 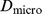 and noise vector 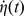 (Fig. 7 B). On long timescales of several 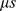, the MSD became sub-linear and converged to the same constant for all system setups (Fig. 7 A). This is a finite-size effect, i.e. the MSD is limited by the size of the container. While a control simulation without repulsive particle-particle interactions (Fig. 7, black lines) only showed these two phases, all setups, where particles had repulsive interactions, exhibited a third phase at intermediate timescales (Fig. 7B). In this intermediate phase, the MSD showed a linear behavior corresponding to a smaller diffusion constant 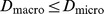. The larger the occupied volume fraction by particles in the setup, and thus the denser the system, the smaller 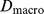 did become (see Fig. 7). This decrease of the effective diffusion constant 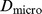 to 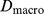 can be explained by crowding: in the first few simulation timesteps, most particles can move along a free path, thus giving rise to the intrinsic diffusion constant 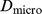. After the time required to move the mean free path length however, particles start to collide with each other. On average, this crowding slows down the diffusion, giving rise to the effective diffusion constant 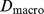. Note that the effective diffusion constant 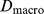 is the quantity that is accessible by experiments such as FRAP.

Figure 7C shows the effective diffusion constants 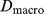 obtained for both particle types, 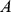 and 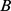, using different system densities. In the “black” scenario (no repulsion), 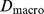 equals 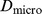. Thus comparing the sizes of colored to the black bars allows to estimate the “slow down” effect of crowding on diffusion. In the most dense situation (50% occupied volume fraction), the diffusion constant of the small particles 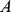 decreased to 66% of its original value (from 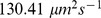 to 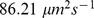). For the larger particle type 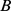, the diffusion constant decreased to 40% of its original value (from 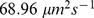 to 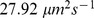). Note that the particles, in the setup without inter particle repulsion potentials, were still exposed to the confining container potential. This potential already slowed down the diffusion constant from the free diffusion case from  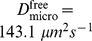 to 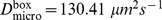 for particle type 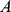 and from 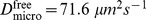 to 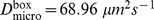 for particle type 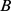.

### Reaction Kinetics and Crowding

To validate the ReaDDy implementation of reaction kinetics and investigate the effect of crowding on reactions, we compared the kinetics of the bimolecular reaction (Eq. (5)) in the binary particle mixture with 30% occupied volume fraction in the following three cases: An ODE solution, a ReaDDy simulation without particle repulsion potentials, and a ReaDDy simulation including repulsion potentials.

In order to compare the ReaDDy simulation to the ODE simulation, the ReaDDy microscopic reaction rates need to be set such, that they give rise to the macroscopic reaction rates used in the ODE. The diffusion constants and reaction radii given in Table 1 give rise to a diffusional encounter rate of 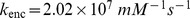 (Eq. (6)). Using Eq. (7), these quantities were used together with the macroscopic reaction rate to calculate the corresponding microscopic reaction rate.

**Figure 8:**
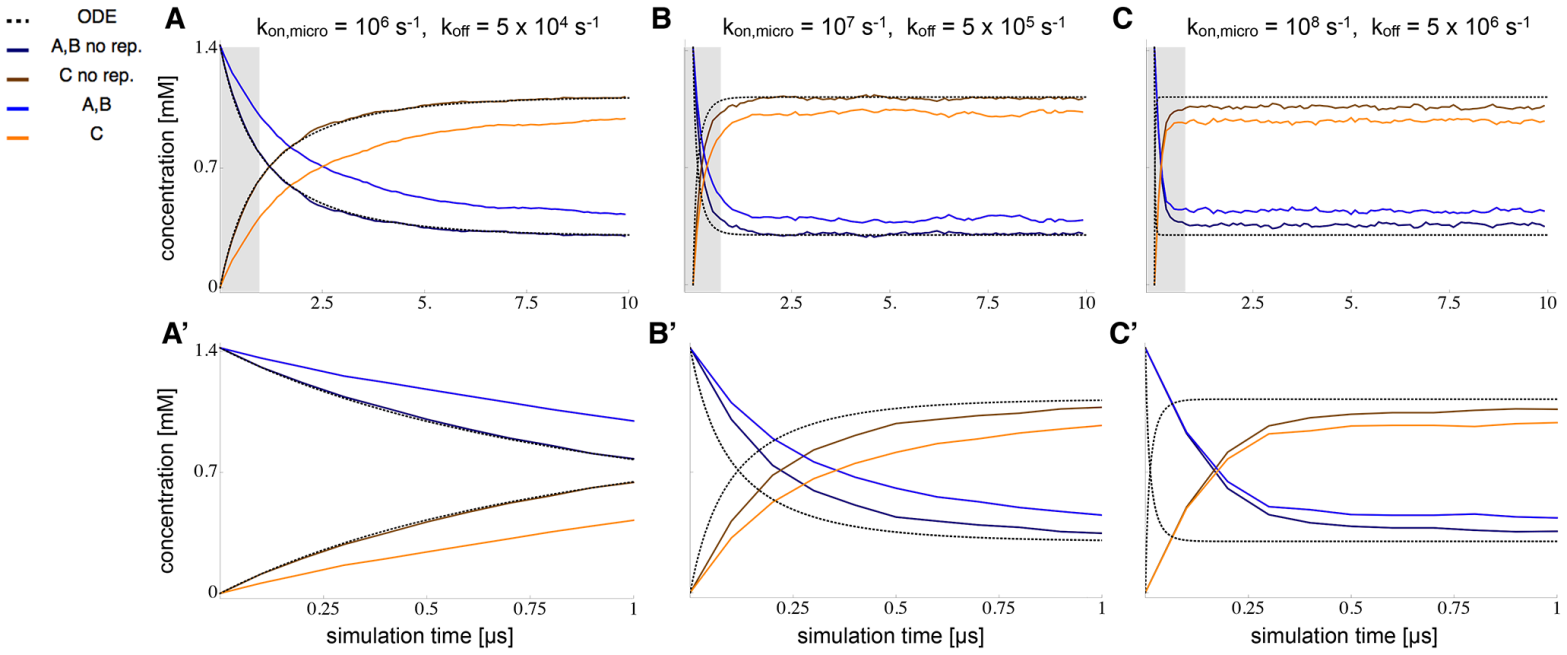
Comparison of ODE Reaction Kinetics with ReaDDy Simulations at Different Reaction Rates. Time-dependent concentrations of 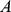, 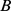, 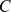 species are reported for reaction 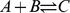 in the 30% benchmark system. ODE solutions (dotted lines) are compared to ReaDDy simulations (colored lines), simulated once with (light blue, orange) and without (dark blue, brown) particle repulsion. The reaction is simulated at different rates: **A:** 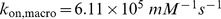, **B:** 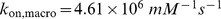 and **C:** 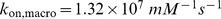 (see figure for values of the microscopic rates). **A’**, **B’** and **C’** depict magnifications of the gray areas in A, B and C. At condition A, reactions are slow enough to allow particles to mix well between reactions. If particle-particle repulsion potentials are switched off, the ODE solution agrees with the ReaDDy solution. If particles do have repulsion potentials, the corresponding minimal distance between reacting particles reduces the volume of space in which a reaction can take place. This effectively lowers the reaction probability and thus slows down the reaction. At conditions B and C the reaction rate is so fast that the well-mixed assumption of the system breaks down. Hence the ODE solution can no longer accurately predict the evolution of the reaction.

The results of the ReaDDy simulation and the ODE kinetics are first compared in a situation where the ODE assumptions are valid. An ODE scheme assumes the reaction container to be well-stirred at each point in time. Thus the reaction rates of the reaction were chosen small enough, compared to diffusion times, such that the system had enough time in between reaction events to equilibrate again: 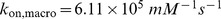 (leading to 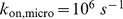 for the ReaDDy simulation) and 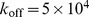. The comparison between the ODE solution and the ReaDDy simulation results show excellent agreement (compare the dark blue and brown lines (ReaDDy) and the dashed black line (ODE) in part A and A′ of Figure 8). It demonstrates that the ReaDDy reaction kinetics implementation is valid.

When reactions are fast compared to diffusion times, the system does not have enough time to equilibrate particle positions before the next event happens. In this situation, the ODE scheme is no longer valid and differences between the ODE solution and the explicit ReaDDy solution are expected. To simulate this scenario, we have increased the reaction rates by one and two orders of magnitude. The results, shown in parts B and C of Fig. 8, demonstrate that the ODE and ReaDDy solutions indeed differ. This difference increases when rates become faster, thus deviating stronger from the well-mixed assumption of the ODE. The ReaDDy solutions reached the equilibrium state slower than the ODE solution because the time to form an encounter complex by diffusion becomes relevant in this scenario.

At high reaction rates, ODE and ReaDDy solutions differ not only in the time-course of the reaction, but also in their stationary concentrations. While the off-rate in scenario B was sufficiently slow, compared to diffusion, to allow a quasi well-mixed state after some equilibration time, this is no longer possible in C. As diffusion becomes limiting in the formation of 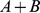 encounter complexes, the effective association rate decreases, resulting in a decreased equilibrium concentration of the product 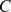.

In order to study the effect of crowding on the reaction kinetics, the particle repulsion potentials for the different setups were switched on (see Fig. 8, light blue and orange lines). Since the reaction radii were not changed, this resulted in an effective decrease of overlap volume in which reactions can occur. The resulting reduction in the reaction rate is visible in all three setups, A, B and C. The relaxation of the concentration to the steady states is now slower. The equilibrium concentration is altered as well because of three effects: 1) The decreased reaction volume of 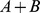 particles leading to a decrease in the effective association rate. 2) The (crowding induced) decreased macroscopic diffusivity of 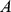 and 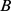 causes both 2a) a reduced encounter rate of 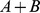 on larger scales and 2b) an increased encounter rate of 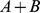 on smaller scales. In 2a), it takes the slower diffusing particles longer to find each other, leading to a decreased production of 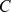. In 2b), 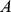 and 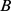 particles, that just emerged from a dissociated 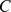 particle, can no longer diffuse away from each other that quickly. This results in a higher probability to react back to 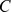 again, in effect stabilizing 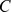. Overall, the effects reducing the effective 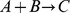 association rate dominate in the present setup, leading to an overall higher educt and lower product concentration in the equilibrium. Please note that the current implementation of ReaDDy does not strictly fulfill detailed balance for reversible reactions. The detailed balance constraint, which is planned for future implementations, will also affect the equilibrium in the present example.

## Using ReaDDy

In order to use ReaDDy, it is sufficient to download the binary from https://simtk.org/home/readdy. It is published Open Source under the BSD 3-Clause License.

Since ReaDDy is Java based, there is no need to compile the source code. The recommended java version is Java SE 6 or newer. To facilitate the start for new users, we developed a tutorial for ReaDDy which can be found on the same website. It contains the ReaDDy binary, together with a tutorial script and predefined input files. During the tutorial, the user is lead through the features of ReaDDy in a step by step fashion on the example of the vesicle fusion process in synapses [6] (Figure 2A shows a snapshot from the final tutorial level).

### Possible Biological Applications

ReaDDy can be applied to model a large spectrum of biological reaction-diffusion systems. Examples include the rod cell phototransduction module [74] and the synaptic vesicle fusion module [75].

The classical model of rod cell phototransduction assumes the rhodopsin (R) photoreceptor molecules to be arranged as freely diffusing, uniformly distributed monomers. Recent results however, have reported R-dimers, racks of R-dimers arranged in ‘paracrystaline’ structures [76], centered R-patches [77] and immobile R-fractions [78]. The diffusional motion of R and of the second messenger molecules, the G-proteins (G), is quite different in some of these scenarios [79]. This may have a non-negligible influence on the output of the photo-activation cascade in which the reaction of R and G is the initial step. With ReaDDy, pairing interaction potentials with reactions in a diffusion simulation, the signal response behavior can now be investigated in these different scenarios.

Vesicle exocytosis relies on the aggregation of enough SNARE proteins to trigger vesicle fusion with the membrane. These proteins have been found to form clusters [17], [19], [80]. With ReaDDy, using attractive interactions between them, the formation and dynamic evolution of clusters can be studied. In subsequent steps, reactions can be included, leading to a model for vesicle fusion and neurotransmitter release. Modeling these processes at microscopic detail is relevant because of the highly scaffolded environment at and around active zones, e.g. through Bruchpilot [81].

### Modeling in ReaDDy

Vesicle exocytosis is used as an example to demonstrate the modeling of biological systems in ReaDDy. Modeling can be done at various levels of detail. Snapshots of the final models are depicted in Figure 2 A and B. The first of the two models is part of the ReaDDy tutorial, providing a step by step introduction for new users.

Fig. 2A shows a setup for the vesicle fusion module [75] in the synaptic vesicle cycle [6]. Vesicle fusion was modeled on a coarse level, including SNARE proteins, vesicles, calcium channels and calcium ions. All vesicles and biomolecules are modeled as single spherical particles with specific properties e.g. radius and diffusion constant. Potentials from the predefined ReaDDy potential library, define the simulation geometry: A disk shaped membrane potential constrains the membrane-bound SNARE proteins, and the calcium channel to a 2D disk shaped surface. A cylindrical potential on top of the disk prevents the cytosolic particles (vesicles and calcium ions) from escaping. Other predefined potentials are used and parameterized for particle interactions in space: Excluded volume of particles is realized by harmonic repulsion potentials that prevent particle-particle penetrations. Clustering of SNARE proteins is modeled by appropriate attractive potentials [19], [80] that on one hand prevent particle penetration but exert attractive forces to nearby particles of the right type on the other. Finally, reactions govern the dynamics of the model (See Text S1, Table S2 and Table S3): *type conversion* reactions govern the switch between the open and closed form of syntaxin, *fusion* reactions make SNAP-25 and syntaxin form a SNARE complexes with a certain probability upon collision. Other *fusion* reactions between SNARE complexes and synaptic vesicles model the binding of SNARE complexes to vesicles. Vesicles change their type upon fusion with SNARE complexes and become membrane bound. When three SNARE complexes have bound a vesicle, the fourth one leads to a fusion ready vesicle. To model the vesicle fusion with the membrane, which sets the SNARE complexes free again, a *fission* reaction is used: It replaces the vesicle particle by two SNARE-complex dimers that themselves react in a *fission* reaction immediately, eventually handing four individual SNARE complexes. In this way, higher order reactions (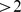) can be modeled. Finally, *type change* reactions govern the switch between open and closed form of the calcium channel, that may create calcium particles in *birth* reactions in its open form. This qualitative model is part of the ReaDDy tutorial that familiarizes the user with ReaDDy’s functionalities by constructing and simulating this model step by step.

Fig. 2B demonstrates the possibilities to describe complex geometries of biomolecules with ReaDDy: a synaptic vesicle’s diffusional approach to the membrane and its membrane association is taken as an example. The system includes the same molecules as the coarse system above. The modeling of the molecules is different however. SNARE proteins now consist of multiple particles, linked together via harmonic potentials to mimic their chain-like form. In ReaDDy, groups of particles can be defined that contain template coordinates for the particles and potentials between them. This facilitates modeling multiple copies of detailed proteins. To model the membrane bound parts of the proteins, special anchor particles are used. For syntaxin, anchors are subjected to a 2D membrane potential, forcing anchors to stay within the plane but repelling the other protein particles. Anchors of vesicle bound proteins (synaptobrevin and synaptotagmin) are subjected to a spherical vesicle membrane potential that constrains their diffusional motion to its surface. The spherical membrane potential itself represents the synaptic vesicle. To model its diffusion, the center of the spherical potential diffuses, causing the potential to move, dragging the molecules with it by their anchors.

## Conclusions and Outlook

In this work the software package ReaDDy was introduced. ReaDDy allows a microscopic, particle-based reaction-diffusion simulation to be combined with particle interaction potentials.

Starting from theoretical concepts, the derivation of the algorithm behind ReaDDy was presented. Details about its implementation, the software architecture and ReaDDy’s performance were given. Sections about parametrization of ReaDDy-simulations and their validation against an ODE model showed, that the ReaDDy implementation is correct. These sections may also provide a guide for ReaDDy-users to parametrize and validate their own simulations. To facilitate the start of simulating and developing in ReaDDy, the software is Open Source (BSD 3-Clause), freely available online (https://simtk.org/home/readdy) and is equipped with a step by step tutorial.

ReaDDy can be seen as a classical reaction-diffusion simulation including potential based particle-particle and particle-geometry interactions. These are crucial to simulate crowding effects on a microscopic level. It has been demonstrated before, both in silico and in experiments, that crowding influences molecular diffusion [13], [14], [27], [82]–[85]. It has also been shown, that crowding thereby greatly affects the kinetics of molecular reactions [86]–[89]. ReaDDy offers the opportunity to study both effects at the same time. It was observed that crowding changes particle diffusion, in turn changing the association rate of encounter complexes, the prerequisite of a reaction. Note that in concrete biological applications, the effective interaction potentials between biomolecules are not a priori known. A way to go beyond a rough guess of the interaction potential is to employ detailed MD simulations of individual biomolecular complexes. Using enhanced sampling methods such as umbrella sampling [90] or metadynamics [91] the potential of mean force of the interactions between individual biomolecules can be computed [92]. These results can be used to design interaction potentials in ReaDDy, making efficient simulations of large-scale mixtures of these biomolecules available, which would not be feasible in MD.

We further observed that the inclusion of particle-particle interaction potentials in a microscopic reaction-diffusion system extends beyond the current theory. The derivation of a microscopic reaction rate 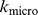 from an experimentally derived macroscopic reaction rate 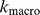 and diffusion parameters is based on the assumption, that the total volume of both interacting particles is available as reaction volume [60]. Particle repulsion potentials reduce this reaction volume by preventing particle penetration, thereby effectively reducing the reaction rate. An extension of the current theory is necessary, that allows the excluded volume effects from particle-particle interactions on the magnitude of 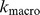 to be taken into account. Development of such a theory can be guided by ReaDDy, which can generate reference solutions. Even without a theory available, the desired value of 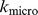 can now be pragmatically obtained by sampling different parameter values and then choosing that which yields the correct macroscopic rate.

ReaDDy has been designed to be expandable and to encourage the implementation of new modules. Separating the BD-*Core Module* from the *Group/Reaction Module*, already anticipates the intended parallelization of the *Core*. For example, high-performance parallel codes that are able to simulate particle diffusion without reactions may be employed here.

We are confident that ReaDDy will prove to be a valuable tool to simulate cellular processes that rely on reaction-diffusion dimensions and require a high degree of realism. This is especially true for processes that are affected by crowding and involve species with small copy numbers. Processes of this type include the synaptic vesicle cycle [6], the Rod cell photo activation cascade [74] and many others. Essentially, as Zimmermann and Trach showed experimentally [73], Goodsell showed with his illustrations[93]–[95] and Elcock and Skolnick with their simulations [12], [14]: the cell is a very crowded, compartmentalized and heterogeneous environment with complex interactions between molecules. ReaDDy appears to provide the appropriate level of model and simulation to accompany the insights into this complexity that are revealed by modern experiments.

## Supporting Information

Text S1 **This text provides details about probing stationary distributions with a Monte Carlo (MC) scheme and further implementation details of ReaDDy.** These include efficient neighbor calculation, input file organization and implemented reaction-types.
(PDF)
