# Supplementary material for: ReaDDy - A Software for Particle-Based Reaction-Diffusion Dynamics in Crowded Cellular Environments
Source: PLoS One. 2013 Sep 11;8(9):e74261. doi: 10.1371/journal.pone.0074261 (PMC3770580; doi:10.1371/journal.pone.0074261)
Supplement: Text S1 — This text provides details about probing stationary distributions with a Monte Carlo (MC) scheme and further implementation details of ReaDDy. These include efficient neighbor calculation, input file organization and implemented reaction-types. (PDF) [file pone.0074261.s001.pdf]

Supporting Information
-
ReaDDy - a Software for Particle-Based Reaction-Diffusion Dynamics in Crowded Cellular Environments

Johannes Schöneberg and Frank Noé

Text S1

Markov Chain Monte Carlo (MCMC) Sampling Scheme for Probing the Stationary Distribution of a Particle Composition

In absence of reactions, the particle composition will not change and the only dynamical process in the system is diffusion of particles, driven by thermal motion. This dynamics is in equilibrium, i.e. it has a well-defined stationary distribution, given only by the potential (manuscript Eq. (8)). In order to have an independent reference to test the implementation of our dynamics, we specify a Monte Carlo (MC) procedure for sampling $p(\mathbf{x})$:

Algorithm S 1 Monte Carlo Sampling Scheme for the Stationary Distribution.

---

1. Start with time $t = 0$, an initial particle configuration $\mathbf{x}_{t=0}$ and an initial energy $E_{t=0}$ of $\mathbf{x}_{t=0}$.
2. Repeat for N steps (total simulation time $N\Delta t$):
   (a) For each particle p with position x_t^p, generate a new random position $\hat{x_t^p}$ with $x_t^p - \hat{x_t^p} \in [0, \sqrt{2D_p\Delta t}]$. This displacement scheme mimicks Brownian motion of particles to be more comparable to our Brownian dynamics simulator. Any other random displacement would also be valid for the MC algorithm.
   (b) Compute the energy E_t' of the new configuration $\mathbf{x}_t' = \mathbf{x}_t \backslash \{x_t^p\} \cup \{\hat{x_t^p}\}$ using the particle repulsion potentials and compute $\Delta E_t = E_t' - E_t$.Accept the new particle position with probability $min\left[1, exp(\frac{\Delta E_t}{k_B T})\right]$ with Boltzmann constant k_B and temperature T.

A separate ReaDDy Core implements this scheme.

Efficient Neighbor Calculation

In particle dynamics simulations the task of calculating neighbors of particles occurs very frequently. All particle-particle interactions such as particle repulsion or reactions between particles are based on the spatial proximity of particles and therefore require the distances between particles to be known.

The naive approach to this problem would be to recalculate all pairwise particle distances in each time step. However this approach would have runtime complexity of $O(n^2)$ where n is the number of particles. This would dominate the runtime of the overall simulation that otherwise only consists of algorithms with linear complexity $O(n)$.

For most practical purposes, particle interactions are limited to a certain interaction distance. The longest-range molecular interactions are electrostatic interactions which reach a few nanometers (1-2 nm in the cytosol, somewhat longer along the membrane). Thus, when simulating signal transduction events on the resolution of one or a few particles per protein, one is faced with computing only nearest-neighbor interactions. In the reaction and interaction schemes shown here, this is implemented by the fact that interactions are only active below a certain distance r_i or r_c.

Reducing distance calculations to nearest-neighbor distance calculations permits to avoid the n^2 runtime and to achieve linear runtime. Various approaches exist for efficient neighborlisting e.g. Verlet Lists. Here, we implement a neighbor lattice approach. A three-dimensional lattice is set up to dissect simulation space, with a box spacing r_{box} equal to the maximum interaction distance in the system: $r_{box} = 2\,max(r_i,\, r_c|', \forall r_i\, \forall r_c)$. To list all possible interaction partners of a certain particle p, we first locate it's grid box, and then iterate over all particles in the adjacent boxes for potential candiates. By construction of the grid, all particles not within these boxes are too far away from p for any interaction.

In the theoretical worst case, all particles are placed in the same box and the lattice neighbor search performs not better than $O(n^2)$. In practice, however, when particles do have repulsive potentials, their density is limited and thus the number of particles that can fit in a $3 \times 3 \times 3$ box environment is limited to a certain maximum m. In this case the complexity of the neighbor search is at most $O(mn)$, i.e. linear in n. In many practical cases, the typical number of particles in a $3 \times 3 \times 3$ box is very small.

Input File Organization

Input-type	Parameters	Topology
**global**	`param_global.xml`	
**reactions**	`param_reactions.xml`	
**particles**	`param_particles.xml`	`tplgy_particles.xml`
**groups**	`param_groups.xml`	`tplgy_groups.xml`
**potentials**		`tplgy_potentials.xml`

Table S 1: **ReaDDy Input File Organization.** The ReaDDy input module operates on seven distinct input files that serve as input sources for the five different input types of ReaDDy. Each input typ is associated to either or both input file types: 1) a parameter file (prefix 'param_') containing general input data about the simulation itself or classes of objects therein (e.g. diffusion constant of a particle type), 2) a topology file (prefix 'tplgy_') containing specific input data about objects within a simulation (e.g. initial coordinates for a given particle).

In the following, the five input types and their related input files are shortly discussed. Please be also referred to the ReaDDy-Manual for a more detailed decription. The general input files are indicated with the prefix '**param_**' in front of their filename and contain data related to classes of objects in the simulation e.g. the 'param_particles'-file contains the different particle types with their associated collision and interaction radii and diffusion constants that apply to all 'objects' of this particle type in the simulation. The specific topology related input files carry the prefix '**tplgy_**' for topology in front of their filename. These files contain data related to the initial configuration of actual objects in the simulation e.g. the 'tplgy_particles'-file defines the initial positions of all particles.

(1) The **'global'** input type refers to all parameters that govern the simulation globally. Input data like the simulation timestep Δt, the simulation temperature, the number of iterations and the boundary of the simulation environment are set here. But also some analysis features can be specified here e.g. the output format of the output trajectory and its frame rate.

(2) Also the **'reactions'** input type only consists of a 'param_' file: In 'param_reactions.xml' each reaction can be defined via its reaction type, its set of educts, its set of products, its $k_{on,micro}$ and its $k_{off,micro}$. In ReaDDy, all reactions are assumed to be bidirectional e.g. the definition of a reaction includes its forward and backward direction. If a unidirectional reaction is intended, one of the $k's$ can be set to 0. All reactions currently implemented in ReaDDy are explained in more detail in the supplementary section 'Reaction Types in ReaDDy'.

(3) The **'particles'** input type governs all data that is related to particles.
In 'param_particles.xml' the particle types can be defined that are usable in the subsequent simulation run. All parameters that are related to these particle types, e.g. that are equal for all particles ('objects'), like the diffusion constant D, the collision radius r_c and the interaction radius r_i, are specified here. To specify collision and interaction radii in more detail, a map datastructure is provided in the file that allows to assign specific radii to specific types of particle interactions e.g. assign for particle type A a different r_c for an interaction with type B than with type C. Parameters that may vary from individual particle to individual particle, like the particle id, the particle type and the particle coordinates are specified in 'tplgy_particles.xml'.

(4) The **'groups'** input type contains all particle group related information. The structure with the 'param_groups.xml' file and the 'tplgy_groups.xml' file is the same as the 'particles' input type. The 'param_' file contains a template for each group type, together with information about its building blocks and the potentials that govern the interactions between these blocks. The 'tplgy_' file defines the initial group configuration for the simulator e.g. which groups are present and which particles belong to which group.

(5) The final **'potentials'** input type is responsible for potential input specifications. Currently, all potentials that can be used in ReaDDy are Java implementations of the 'IPotential1' or the 'IPotential2' interface, representing potentials of order 1 and order 2 respectively. This existing potential library is accessible by the user via the 'tplgy_potentials.xml' file. There, the desired potentials are chosen and defined. Please refer to the ReaDDy-Manual for the list of currently available potentials and their input specifications.

Concearning the input file organization, please also refer to Table S1.

Reaction Types in ReaDDy

Table S2 and Table S3 include all reaction types that can be defined in ReaDDy, based on the concept that the maximal order of a reaction is order two. Table S2 refers to the particle related reactions that can occur between two particles. Table S3 refers to the group related reactions that can occur between groups. To define any of the depicted reactions in 'param_reactions.xml', the type must be specified between the type tags e.g. `<type>enzymatic</type>` for an enzymatic reaction and the educts and products have to be specified according to the ordering in the 'symbol' column. In ReaDDy, all reactions are assumed to be bidirectional. This means the definition of a 'creation' reaction implies that the forward reaction will be an actual 'creation' reaction, while the backward reaction, a 'decay' in this case, will be defined automatically for the backward direction. For the case of 'creation' and 'decay', both reaction types are forward and backward reaction of the same bidirectional reaction scheme and do only differ in their declaration. This applies only for the asymetric bidirectional reactions. E.g in a symetric 'enzymatic' reaction, both forward and backward reactions have the same microstructure (indicated by 'back & forth' in the direction column). The table lists all theoretically possible reactions up to order two. ReaDDy however currently only implements reactions that actually change the particle configuration. For that reason, the 'nothing', 'identical' and 'double identical' reactions are not implemented but still appear in this table for completeness.

ID	Equation	Type	Dir. Sym.	Direction
/	0→0*	nothing	$\leftrightharpoons$	back & forth
0	0→x	creation	$\rightharpoondown$	forth
1	x→0	decay	$\leftharpoonup$	back
2	0→xy	doubleCreation	$\rightharpoondown$	forth
3	xy→0	annihilation	$\leftharpoonup$	back
/	x→x*	identical	$\leftrightharpoons$	back & forth
4	x→y	typeConversion	$\leftrightharpoons$	back & forth
5	x→xy	birth	$\rightharpoondown$	forth
6	xy→x	death	$\leftharpoonup$	back
7	x→yz	fission	$\rightharpoondown$	forth
8	yz→x	fusion	$\leftharpoonup$	back
/	xy→xy*	doubleIdentical	$\leftrightharpoons$	back & forth
9	xy→xz	enzymatic	$\leftrightharpoons$	back & forth
10	wx→yz	doubleTypeConversion	$\leftrightharpoons$	back & forth
11	x|z→x|x,y|y	idConservingBirth (id|type)	$\rightharpoondown$	forth
12	x|x+y|y→x|z	idConservingDeath	$\leftharpoonup$	back

Table S 2: **Particle Related Reactions.** All particle related reactions that are possible and implemented in ReaDDy. *: Reactions that do not change the particle configuration (ID='/') are not implemented, but appear in this table for completeness.

ID	Equation	Type	Dir. Sym.	Direction
100	p_1p_2→g	group	$\rightharpoondown$	forth
101	g→p_1p_2	ungroup	$\leftharpoonup$	back
102	g→h	gTypeConversion	$\leftrightharpoons$	back & forth
103	g→hi	gFission	$\rightharpoondown$	forth
104	hi→g	gFusion	$\leftharpoonup$	back
105	gh→gi	gEnzymatic	$\leftrightharpoons$	back & forth
106	gh→ij	gDoubleTypeConversion	$\leftrightharpoons$	back & forth

Table S 3: **Group related reactions.** All group related reactions that are implemented and possible in ReaDDy.
